# Working Memory Requires a Combination of Transient and Attractor-Dominated Dynamics to Process Unreliably Timed Inputs

**DOI:** 10.1038/s41598-017-02471-z

**Published:** 2017-05-30

**Authors:** Timo Nachstedt, Christian Tetzlaff

**Affiliations:** 1 Third Institute of Physics, Universität Göttingen, 37077, Göttingen Germany; 2 Bernstein Center for Computational Neuroscience, 37077 Göttingen, Germany; 3 Max Planck Institute for Dynamics and Self-Organization, 37077 Göttingen, Germany

## Abstract

Working memory stores and processes information received as a stream of continuously incoming stimuli. This requires accurate sequencing and it remains puzzling how this can be reliably achieved by the neuronal system as our perceptual inputs show a high degree of temporal variability. One hypothesis is that accurate timing is achieved by purely transient neuronal dynamics; by contrast a second hypothesis states that the underlying network dynamics are dominated by attractor states. In this study, we resolve this contradiction by theoretically investigating the performance of the system using stimuli with differently accurate timing. Interestingly, only the combination of attractor and transient dynamics enables the network to perform with a low error rate. Further analysis reveals that the transient dynamics of the system are used to process information, while the attractor states store it. The interaction between both types of dynamics yields experimentally testable predictions and we show that this way the system can reliably interact with a timing-unreliable Hebbian-network representing long-term memory. Thus, this study provides a potential solution to the long-standing problem of the basic neuronal dynamics underlying working memory.

## Introduction

Humans and animals continuously receive information conveyed by stimuli from the environment. To survive, the brain has to store and process this stream of information which is mainly attributed to the processes of working memory (WM^1, 2^). These two distinct abilities of WM, to store and to process information, yield a debate about the underlying neuronal network dynamics^3–5^: the network dynamics might either follow (i) attractor or (ii) transient dynamics.

*Attractor dynamics* denotes neuronal network dynamics which is dominated by groups of neurons being persistently active. In general, such a persistent activation is related to an attractor state of the dynamics, with each attractor associated to a specific information content^3, 6–8^. Several experimental and theoretical studies hypothesize that the dynamics underlying WM are dominated by such persistent dynamics^5, 8–10^. In contrast to attractor dynamics, neuronal networks with *transient dynamics* are dominated by an attractor-less continuous flow of neuronal activity across a possibly large neuronal population^11–14^. This type of dynamics implies a high diversity and complexity which is linked by theoretical studies with a large computational capacity required to process information^15–17^. These theoretical studies as well as several pieces of experimental evidence^18–20^ yield the hypothesis that the dynamics underlying WM are dominated by transient dynamics^20, 21^. Thus, although the two hypotheses – attractor or transient dynamics – seem to contradict each other, experimental and theoretical evidence supports both yielding a debate about the neuronal network dynamics underlying WM^5^.

To resolve this contradiction, in this study, we consider the fact that the timing of stimuli received by the WM is highly unreliable. In other words, when interacting with the environment, the WM of humans and animals evidently cannot rely on receiving precisely timed stimuli. For instance, listening to spoken language requires the ability to deal with different and irregular speech rates. The influence of such variance in the stimuli timing on the WM operation has been mainly analyzed on the psychological level^22^ using, amongst others, the so-called *N*-back task. In this task a subject is exposed to a stream of different stimuli^23, 24^. Whenever a new stimulus is presented, the subject has to execute an action which depends on the stimulus presented *N* stimuli before. Therefore, in order to succeed in this task, the subject has to store the information of the last *N* stimuli in its WM. Dependent on the timing of the stimuli, this information has to be continuously updated. Interestingly, whether the stimuli are presented with exact inter-stimulus timing or with unreliable timing *does not* influence the subject’s performance of solving the *N*-back task^22^. This result indicates that the mechanisms implementing WM are robust against variance in the timing of the input stimuli. Based on this experimentally found property of WM, in this study, we investigate under which conditions the dynamics of the neuronal networks underlying WM is able to perform an *N*-back task with the same robustness with respect to variances in the stimuli timing.

**Figure 1:**
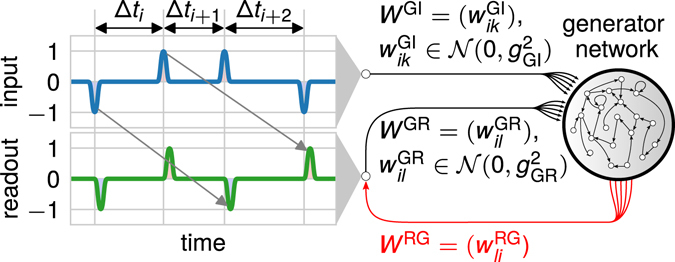
Setup of the benchmark *N*-back task to test the capability of transient networks to cope with variances in the input timings. The input signal is composed of smooth either positive or negative pulses separated by time intervals Δ*t*_*i*_ drawn from a normal distribution with mean *μ*_Δ*t*_ and variance ${\sigma }_{{\rm{\Delta }}t}^{2}$. It is projected into the generator network via a synaptic weight matrix *W* ^GI^ with elements ${w}_{ik}^{GI}$ drawn from a normal distribution with zero mean and variance ${g}_{{\rm{GI}}}^{2}$. The task is to produce an output pulse of defined shape (at the readout neurons) when a new input pulse is presented. The sign of the output pulse depends on the second last input pulse (compare arrows). The readout weight matrix *W* ^RG^ is adapted during learning (red). The resulting readout signal is fed back into the network with a weight matrix *W* ^GR^ with elements ${w}_{il}^{GR}$ drawn from a normal distribution with zero mean and variance ${g}_{{\rm{GR}}}^{2}$.

**Figure 2:**
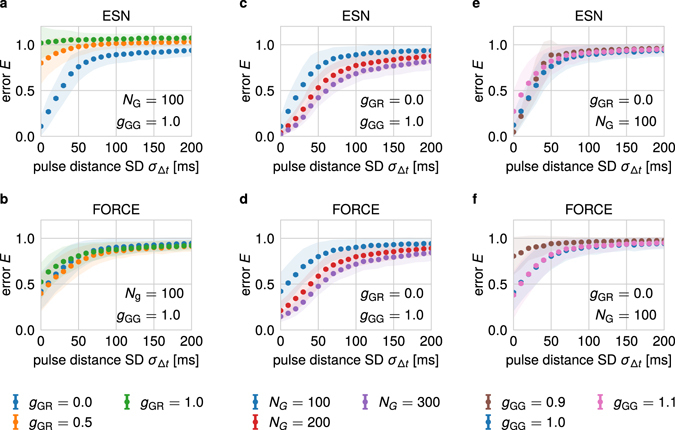
Influence of variances in input timings on the performance of the transient network. The mean normalized readout error *E* (see Methods) for the benchmark task depicted in Fig. 1 increases with larger standard deviation *σ*_Δ*t*_ of the interstimulus intervals of the input stream independent of the used parameters. In (**a**,**c**,**e**), the network is trained using the echo state network approach (ESN). In (**b**,**d**,**f**), the FORCE-learning method is employed. Every data point represents the mean of 1000 network instantiations. The shaded area indicates the standard deviation of the respective error distribution. The error bars show the standard error of the mean. If for one instantiation the error after training is larger than 1.5, we consider the respective training procedure as not converged and exclude it from the mean. (**a**,**b**) The network is trained with three different values of the standard deviation *g*_GR_ of the feedback-weights from the readout neurons to the generator network. For both training methods, increasing *g*_GR_ also increases the error *E* for a given value of *σ*_Δ*t*_. The constant parameters are *N*_*G*_ = 100 and *g*_gg_ = 1.0. (**c**,**d**) Networks of different sizes, i.e. different values of *N*_*G*_, are trained to perform the benchmark task. While larger networks perform better for a given value *σ*_Δ*t*_, they qualitatively show the same strong sensitivity to variances in input timings. The constant parameters are *g*_GR_ = 0 and *g*_gg_ = 1.0. (**e**,**f**) The influence of different values *g*_GG_ of the internal weights of the generator network is investigated. Neither increasing nor decreasing of the critical value *g*_GG_ reduces the error significantly. The constant parameters are *g*_GR_ = 0 and *N*_*G*_ = 100.

**Figure 3:**
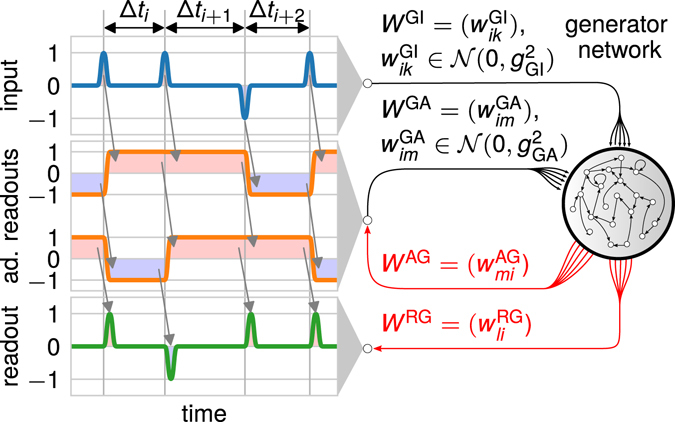
Setup of the benchmark *N*-back task to test the influence of additional, specially-trained readout neurons to cope with variances in the input timings. The input signal as well as the target signal for the readout neuron are the same as before (Fig. 1). Additional neurons, which are treated similar to readout units, are introduced in order to allow for storing task-relevant information. These additional neurons (ad. readouts) have to store the sign of the last and second last received input pulse as indicated by the arrows. The activities from the additional neurons are fed back into the network with weights ${w}_{im}^{{\rm{GA}}}$ drawn from a normal distribution with zero mean and variance ${g}_{{\rm{GA}}}^{2}$ basically extending the network. Synaptic weights adapted by the training algorithm are shown in red. The feedback from the readout neurons to the generator network is set to be zero (*g*_GR_ = 0).

**Figure 4:**
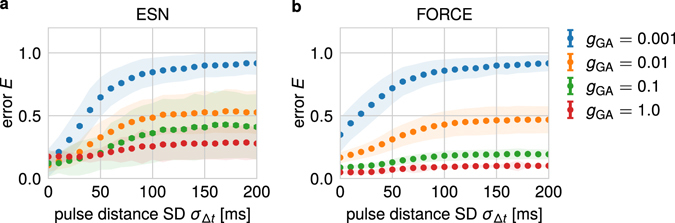
Influence of variances in input timings on the performance of the network with specially-trained neurons. The normalized readout error *E* of a network with specially-trained neurons decreases with larger values of the standard deviation *g*_GA_ determining the feedback between specially-trained neurons and network. If this standard deviation equals 1, the error stays low and becomes basically independent from the standard deviation *σ*_Δ*t*_ of the inter-pulse intervals of the input signal. (**a**) ESN approach; (**b**) FORCE-method.

**Figure 5:**
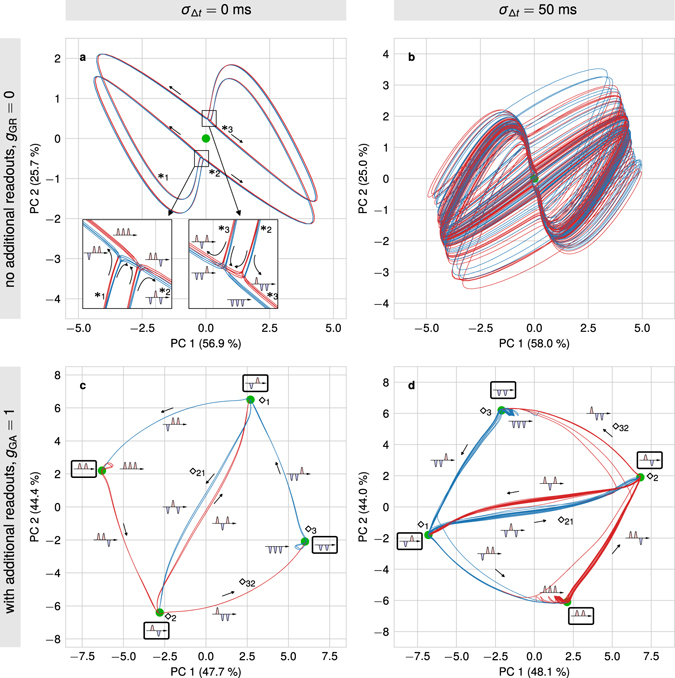
Neural network dynamics during performing the benchmark task projected onto the first two principal components. Trajectory sections which should trigger a positive pulse at the readout units are drawn in red while those which should trigger a negative response are shown in blue. The small arrows indicate the direction in which the system flows along the trajectory. The small pictograms indicate the recent history of the input pulses along the time axis. Green dots indicate attractor states (manually added). (**a**) The network without additional readouts (Fig. 1) stores the history of stimuli on transients. (**b**) By introducing variances in input timings, these transients smear impeding a proper readout. (**c**) The additional readouts (or specially-trained neurons; Fig. 3) “structure” the dynamics of the system by introducing several attractor states each storing the history of the last two stimuli. (**d**) Even in the presence of timing variances the attractor-dominated structure in phase space is preserved enabling a proper readout. Parameters: mean inter-pulse interval *μ*_Δ*t*_ = 100 ms; (**a**) *g*_*GR*_ = 0, *σ*_Δ*t*_ = 0 ms; (**b**) *g*_*GR*_ = 0, *σ*_Δ*t*_ = 50 ms; (**c**) *g*_*GA*_ = 1, *σ*_Δ*t*_ = 0 ms; (**d**) *g*_*GA*_ = 1, *σ*_Δ*t*_ = 50 ms. Details see Supplementary Discussion S1.

**Figure 6:**
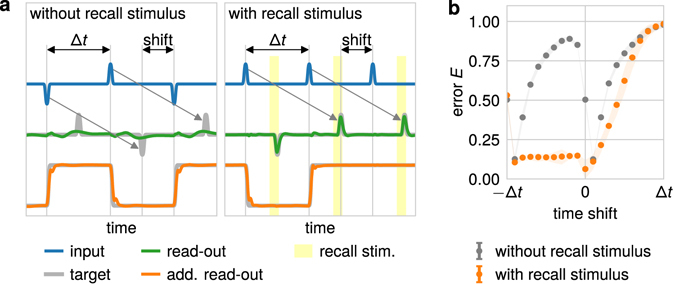
Prediction of the influence of an additional recall stimulus. (**a**) An additional temporal shift is introduced between input and output pulse. In the second setup (lower row) a recall stimulus is applied to the network to trigger the output. This recall stimulus is not relevant for the storage of the task-relevant sign. (**b**) In general the temporal shift increases the error of the system (gray dots; each data point indicates the average over 20 trials) as the system has already reached an attractor state. Introducing a recall stimulus (orange dots) decreases the error for all negative shifts as the system is pushed out of the attractor and the task-relevant information can be read out. This effect diminishes for positive temporal shifts as the system has already forgotten the corresponding information.

First, we investigate a theoretical neuronal network model of WM showing purely transient dynamics – a so called reservoir network^25, 26^ – and test its performance on the *N*-back task. Interestingly, with small variations of the timing of the inputs, such a purely transient system exhibits a very poor performance (Figs 1 and 2). In the next step, we show that the performance of the network increases significantly if the system is directly trained in a supervised manner to maintain the relevant information (Figs 3 and 4). A further analysis reveals that the underlying neuronal dynamics of the trained system are dominated by attractor states which are interlinked by regions of transient dynamics. By comparing these combined dynamics with the dynamics of the purely transient system during performing the *N*-back task, we demonstrate that only this combination of attractor and transient dynamics allows the execution of the task robust against variances in stimuli timings (Fig. 5). In addition, we show that, in general, the attractor states store the task-relevant information while the transient dynamics processes the information (Figs 3 and 6). This yields the prediction that a drop in performance resulting from an additional delay between the current stimulus and the execution of the action can be avoided by introducing another stimulus pushing the system into a transient state (Fig. 6).

**Figure 7:**
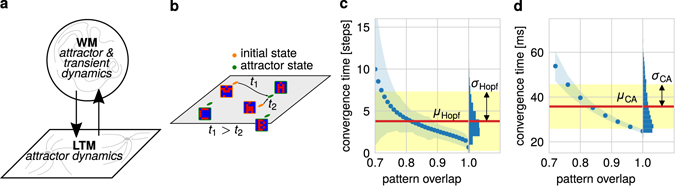
Network models of long-term memory show variances in recall timings. (**a**) As WM and LTM interact continuously, inherent properties of the LTM network influence the function of WM. (**b**) In standard network models of LTM, the recall of a memory representation (e.g., the letter “A”) corresponds to the convergence of the neuronal system dynamics to a previously learned attractor (green dots). The time span required until convergence (“convergence time”; here *t*_1_ and *t*_2_) depends on the initial state of the system or the recall stimulus (differently altered “A”s; orange dots). **(c)** 100 random patterns are stored into a standard Hopfield network^43^ with *N*_Hopf_ = 1000 neurons. Patterns with differences in *d* neurons to one of the stored patterns, corresponding to a pattern overlap of 1 − *d*/*N*_Hopf_, are used as initial states for the system. The number of simulation steps to reach the stored pattern represents the convergence time (dots mark average value over 10000 trials with standard deviation indicated by blue shading). The histogram on the right-hand side illustrates the resulting distribution of convergence times for a uniform distribution of initial pattern overlaps (red line shows mean *μ*_Hopf_ and yellow shading the corresponding standard deviation *σ*_Hopf_). **(d)** The recall convergence times analyzed similar to panel (C) for a self-organizing cell-assembly network^28^. A cell assembly is trained to represent a given input pattern. After training, a similar pattern with an overlap of 1 − *d*/*N*_aff_, is presented to the network (i.e. *d* neurons have a different activity compared to the learned pattern). The time span required to activate 90% of the cell assembly represents the convergence time.

**Figure 8:**
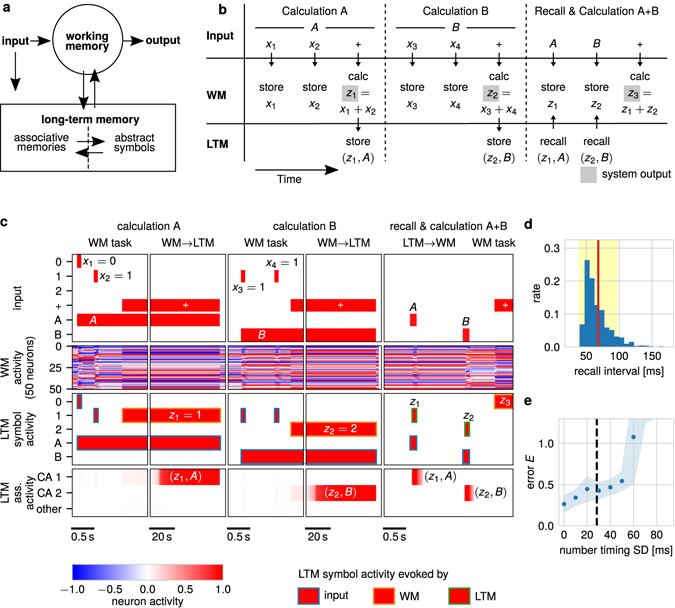
A multi-phase task requires the continuous interaction between WM and LTM. A WM network consisting of attractor states and transient dynamics enables the reliable interaction with an LTM network to solve a complex multi-phase task. Details see main text. (**a**) The external input is projected into WM and LTM. For simplicity, the LTM area is separated into two compartments: the first stores abstract symbols and the second forms input-dependent associations between these symbols. (**b**) Multi-phase task and information flow between input, WM, and LTM. (**c**) External inputs, activities of 10% of the *N*_*G*_ = 500 WM-neurons, activities of the neurons within the LTM symbol area and activities within the LTM association area during the three phases. For the activities in the LTM symbol area, we indicate whether this activation is evoked by the external input, the WM, or from the LTM association area. (**d**) Distribution of the time intervals needed for the recall of the information stored in cell assemblies in the LTM-association area for equally distributed trials with 75% to 100% complete context signals. Here, the recall interval is defined as the time span between the onset of the external context stimulus and the point of time at which more than 50% of the neurons in the LTM-symbol area, representing the recalled number, fire at rates higher than 90% of the maximum firing rate. The standard deviation of this distribution is approximately 28 ms. (**e**) Error of a purely transient network with *N*_*G*_ = 500 neurons performing the summation task (third phase) with only variances in input timings from the LTM recall alone. According to (**d**), this source of unreliability alone already doubles the readout error (dashed vertical line).

Furthermore, besides stimuli from the environment, also stimuli from other brain mechanisms, as long-term memory (LTM), are characterized by unreliable timing. We show that in established theoretical neuronal network models of LTM^27, 28^ the time needed for a cue-triggered recall of stored information varies dependent on the initial conditions of the recall-triggering cue and the neuronal network (Fig. 7). Due to the continuous coupling between WM and LTM, which is fundamental in order to solve complex tasks^29–32^, this variance in recall timings has to be reflected in the dynamics of the WM. Thus, we show that, similar to the *N*-back task, only a neuronal network with a combination of attractor and transient dynamics enables a continuous and reliable coupling between WM and LTM which can be used to solve a complex multi-phase task (Fig. 8). This describes, to our knowledge, the first theoretical model of the functional, dynamic interaction between a WM- and a LTM-network.

## Results

### Reservoir networks are vulnerable to variances in stimuli timings

Stimuli received by the working memory (WM), coming from the environment as well as from the long-term memory (LTM), are characterized by an unreliable timing of their occurrence. Thus, to function in a reliable manner, the WM has to reduce the influence of these timing variances. Within the last years, neuronal networks with purely transient dynamics –so-called reservoir networks^25, 26^ –have been proposed as a theoretical model of WM^5^. A reservoir network consists of a generator network, being composed of *N*_G_ basically randomly connected neurons, which receives temporally varying input stimuli from a set of *N*_I_ input neurons and projects signals to a downstream output layer with *N*_R_ readout neurons. Due to the random connectivity within the generator network, the input stimuli are transformed into a variety of complex traces or, in other words, the inputs are processed in different variants by the network. As a consequence, the readout of a desired processing or target signal requires only the optimization of the weight matrix *W* ^RG^ of the synapses transmitting signals from the generator neurons to the readout neurons. In the following, to ensure generality of our results, the readout weight matrix *W* ^RG^ is optimized by two alternative strategies (for details see Methods section): On the one hand, we use the offline ESN-approach^25^ and, on the other hand, we apply the online FORCE-algorithm^33^. Thus, after optimization, the readout neurons optimally “combine” the signals naturally present in the generator network. Reservoir networks have been shown to posses a high computational capacity as well as a short-term memory capacity^16, 25, 26^ which are the two main components of WM.

We investigate the capability of a standard reservoir network to cope with the described timing variances in a setting similar to the *N*-back task. The neuronal network receives a stream of input stimuli, each of them is a pulse and has either a positive or negative sign (blue line in Fig. 1). At every occurrence of an input stimulus, the network has to produce a non-zero output signal at the readout neuron (green line) of predefined temporal shape (target shape) with a sign equaling the sign of the stimulus received *N* stimuli before (here, *N* = 2; indicated by arrows). Note that even though here the target shape for the output has the same pulsed shape as the input stimuli, the computational capacity of the network allows that it could be of arbitrary shape (see Supplementary Figure S1 for an example with sine-shaped output signals). In general, to solve this *N*-back task, the network has to fulfill two sub-tasks: It has to store the sign of the last two input stimuli (storage of information) and, given the next input pulse, it has to produce an output signal of target shape with the sign equaling the pulse presented *N* stimuli before. The latter depicts the processing of information as the network has to “combine” the stored information (sign) with the transient network dynamics to produce a complex temporal output signal (target shape). Note that the used target shape also implies that the output signal is zero if no input is present. Variances in the timing of occurrence of the input stimuli are introduced by randomly drawing the interstimulus intervals Δ*t*_*i*_ from a normal distribution with mean *μ*_Δ*t*_ and variance ${\sigma }_{{\rm{\Delta }}t}^{2}$.

The performance of a reservoir network instantiation on the *N*-back task is evaluated after the training of its readout weight matrix by calculating the root mean square error *E* determining the difference between target and actual output signal (see Methods). We systematically investigate the influence of the variance in the timing of the input stimuli by varying the standard deviation *σ*_Δ*t*_ of the interstimulus intervals Δ*t* while keeping the mean *μ*_Δ*t*_ constant. For each value of the standard deviation, we average the performance over 1000 different (random) network instantiations. Overall, independent of the training method (ESN as well as FORCE) used for the readout weights, the averaged error 〈*E*〉 increases significantly with increasing values of *σ*_Δ*t*_ until it converges to its theoretical maximum at 1 at about *σ*_Δ*t*_ ≈ 100 ms (Fig. 2). Note that errors larger than 1 are artifacts of the used training method. The increase of the error (or decrease of the performance) with larger variances in the stimuli timings is independent of the parameters of the reservoir network. For instance, we tested the influence of different values of the variance ${g}_{{\rm{GR}}}^{2}$ of the feedback weight matrix *W* ^GR^ from the readout neurons to the generator network (Fig. 2a for ESN and b for FORCE). For the present *N*-back task, feedback of this kind does not improve the performance, although several theoretical studies show^33–35^ that feedback enhances the performance of reservoir networks in other tasks. In contrast, we find that increasing the number of generator neurons *N*_G_ reduces the error for a broad regime of the standard deviation *σ*_Δ*t*_ (Fig. 2c and d). Nevertheless, the qualitative relationship is unchanged and the improvement is weak implying a need for large numbers of neurons to solve this rather simple task for medium values of the standard deviation. Another relevant parameter of reservoir networks is the standard deviation *g*_GG_ of the distribution of the synaptic weights within the generator network determining the spectral radius of the weight matrix^25^. In general, the spectral radius determines whether the network operates in a sub-critical, critical or chaotic regime and also influences the time scale of the reservoir dynamics^33, 36^. Here, we find that both an increase as well as a decrease of *g*_GG_ of about 10% decrease the performance of the system (Fig. 2e,f). Additionally, it turns out that all findings remain valid also when the performance of the network is evaluated in a less restrictive manner by only distinguishing three discrete states of the readout and target signals (Supplementary Figure S2).

In summary, independent of the used parameter values, we find that if the input stimuli occur in an unreliable manner, a reservoir network with purely transient dynamics has a low performance in solving the *N*-back task. This raises doubts about its applicability as a plausible theoretical model of the dynamics underlying WM.

### Specially-trained neurons improve the performance

To obtain a neuronal network which is robust against variances in the timing of the input stimuli, we modify the reservoir network to allow for more stable memory storage. For this, we add (here, two) further neurons to the system and treat them as additional readout neurons by training (ESN as well as FORCE) the weight matrix *W* ^AG^ between generator network and added neurons (similar to the readout matrix *W* ^RG^). Different to the readout neurons, the target signals of the added neurons are defined such that, after training, the neurons produce a constant positive or negative activity depending on the sign of the last or second last input stimuli, respectively (Fig. 3). The activities of the additional neurons are fed back into the reservoir network via the weight matrix *W* ^GA^ (elements drawn from a normal distribution with zero mean and variance ${g}_{{\rm{GA}}}^{2}$) basically extending the generator network. This procedure ensures that the network memorizes the later-on required information^14, 37^. Note that the feedback from the readout neurons to the generator network is neglected (*g*_GR_ = 0).

As above, we evaluate the performance of the extended network while solving the *N*-back task. In general, for a weak feedback from the additional neurons to the generator network (small values of *g*_GA_), larger standard deviations *σ*_Δ*t*_ of the interstimulus intervals Δ*t* result in larger errors *E* (Fig. 4a for ESN and b for FORCE). However, increasing the standard deviation *g*_GA_ of the synaptic weights from the additional neurons to the generator network decreases the influence of the variances in stimuli timings on the performance of the system. For *g*_GA_ = 1.0, the error is only slightly dependent on the standard deviation *σ*_Δ*t*_ of the interstimulus intervals (Fig. 4). The extension of the network by these specially-trained neurons yields a significant improvement compared to the best setup without these neurons (Fig. 2). Please note that this finding also holds for a less restrictive performance evaluation (Supplementary Figure S3). Additionally, the same qualitative finding can also be obtained for significantly larger reservoir networks (Supplementary Figure S4). In the following, we investigate the dynamical principles underlying this increase in performance.

### The combination of attractor and transient dynamics increases performance

Instead of analyzing the complete high-dimensional activity dynamics of the neuronal network, we project the activity vectors onto its two most significant principal components to understand the basic dynamics^38^ underlying the performance changes for the *N*-back task.

For the purely transient reservoir network (without specially-trained neurons; Figs 1 and 2), we investigate the dynamics of the system with *g*_GR_ = 0, *N*_G_ = 100, and *g*_GG_ = 1 as a representative example in more detail (Fig. 5a). The dynamics of the network is dominated by one attractor state at which all neuronal activities equal zero (silent state). However, as the network continuously receives stimuli, it never reaches this state. Instead, dependent on the sign of the input stimulus, the network dynamics runs along specific trajectories (Fig. 5a; red trajectories indicate that the second-last stimulus was positive while blue trajectories indicate a negative sign). The marked trajectory (*_1_ → *_2_ → *_3_) corresponds to a network having recently received a negative and two positive stimuli which now is exposed to a sequence of two negative stimuli (for details see Supplementary Discussion S1). The information about the signs of the received stimuli is stored in the trajectory the network takes (transient dynamics). However, the presence of variances in the timing of the stimuli significantly perturbs this storage mechanism of the network. For *σ*_Δ*t*_ = 50 ms (Fig. 5b), the trajectories storing positive and negative signs of the second-last stimulus cannot be separated anymore. As a result, the downstream readout neuron fails to extract the task-relevant information.

Extending the reservoir network by the specially-trained neurons changes the dynamics of the system significantly (here, *g*_GA_ = 1): The network now possesses four distinct attractor states with specific, transient trajectories interlinking them (Fig. 5c). The marked trajectory (◇_1_ → ◇_21_ → ◇_2_ → ◇_32_ → ◇_3_) corresponds to the same sequence of stimuli as above (for details see Supplementary Discussion S1). Here, the information about the sign of the two last stimuli is stored in the attractor states while the transients, connecting them, are used to process the information and to produce the complex output signal (target shape). Due to the attractor states, which “structure” the dynamics, variance in the timing of stimuli (here, with a standard deviation of *σ*_Δ*t*_ = 50 ms) does not significantly alter the neuronal dynamics (Fig. 5d). The different trajectories and attractor states remain clearly separated. This separation in the presence of timing variance enables the downstream neurons to read out the task-relevant information, which is the dynamical cause of the good performance in the *N*-back task.

Note that, although an attractor state stores information, this information cannot be read out when the output signal has to be time-dependent (non-constant). This includes tasks during which the information has to be read out at specific periods of time and otherwise not. By introducing a time shift between stimulus and information readout (output) even without variances in input timings (constant Δ*t*; Fig. 6), this yields an interesting prediction. A negative shift means that the information has to be read out before the next stimulus is presented. Interestingly, for large negative shifts (≈−Δ*t*), the error is small as the readout is required briefly after the last stimulus and the system is still in a transient state. With smaller negative shifts, the error increases as the system reaches the next attractor and the information cannot be read out anymore in a time-resolved way. Note, however, that the pure information about the sign can be easily read out from the reservoir as done by one of the two specially - trained readout units. This information alone, however, cannot be used to generate a time-resolved signal. The situation changes when we introduce another stimulus with task-irrelevant sign which triggers the output: now, the error is small for all negative shifts as the stimulus “kicks” the system out of the attractor back in a transient state such that the stored information can be read out in a time-resolved way. This does not work for positive shifts as, here, the relevant information is already lost (overwritten by sign of next stimulus).

These results demonstrate that the information is reliably stored in the attractor states of the network dynamics while the information processing (temporally specific readout) happens on the transients. This combination of dynamics also enables the reliable interaction of the WM with other brain mechanisms as shown in the following.

### Attractor and transient dynamics enable continuous interaction with long-term memory

For solving complex tasks humans and animals incorporate several brain mechanisms which yields, amongst others, to a continuous interaction between working memory and long-term memory (LTM)^29–32^ (Fig. 7a). On the one side, information can be transmitted from the WM to the LTM to be stored and to “free” the computational capacities of the WM for processing further information. On the other side, information stored in the LTM can be transmitted back to the WM to be processed. Such interactions imply that the WM has to deal with the inherent properties of the LTM arising from its underlying dynamics. Several experimental and theoretical studies indicate that the neuronal networks implementing LTM are dominated by attractor dynamics^39–42^. Thereby, an attractor state corresponds to a long-term memory representation which is recalled if the network dynamics converges to this state. Note that the convergence time – the time span the system requires to reach the attractor state – is mainly influenced by the recall stimulus and by the initial state of the system (Fig. 7b). As both the recall stimulus and the initial state vary between different recall trials, this variation yields a broad distribution of convergence times (Fig. 7c for the Hopfield model^43^ and Fig. 7d for a Hebbian cell assembly model^28^; please see Methods for more details), which is also found in psychophysical experiments^44^. The width of this distribution depends on the parameters of the system, as the network size^45^, but it always stays larger than zero implying a variance in the convergence times. In other words, if a complex task requires that information has to be recalled from the LTM and transmitted to the WM, this recalled information reaches the WM with unreliable timings.

In the following, we will focus on a complex multi-phase task requiring the interaction between WM and LTM (Fig. 8a,b). For simplicity, we assume that the LTM has already formed representations of abstract symbols as numbers and learning context. Now, the LTM has to form associations between these abstract symbols dependent on external inputs and inputs from the WM. Here, we use a self-organizing cell assembly network to form and store these associations (similar to the system used for Fig. 7d ^17, 28^). The WM receives inputs (external and from the LTM), processes them, and provides the system’s output. The dynamics of the WM are governed by the combination of attractor and transient dynamics described above. This combination enables the WM to deal with the unreliability of the LTM-network to provide the task-dependent information.

The task consists of several phases (Fig. 8b,c): In the first phase (context *A*), the WM-network receives and stores two stimuli (*x*_1_ and *x*_2_) each representing a number, followed by a + -signal instructing the WM to add both numbers (for simplicity we use for all calculations the modulo-three-operator, thus, *z*_1_ = *x*_1_ + *x*_2_ mod 3). The result *z*_1_ is transferred to the LTM which forms a cell assembly storing the association between context *A* and result *z*_1_. Thus, the WM processes the information and the LTM stores the result of this processing for later reuse. This “frees” the WM making its processing capacities available for other tasks, as needed in the second phase (context *B*) during which these capacities are used for performing a second calculation. Similar to the first phase, two numbers are presented (*x*_3_ and *x*_4_) and the WM calculates the sum of them. The result is maintained in the LTM by forming a new cell assembly storing the association between context *B* and result *z*_2_. In the third phase (context *A* + *B*), the information stored in the LTM is transferred back into the WM for further processing. Namely, by externally activating the context signal *A* (*B*), the LTM recalls the stored association and transfers *z*_1_ (*z*_2_) to the WM. Importantly, due to the variance in convergence times of the LTM (Fig. 7d), the WM receives the information *z*_1_ and *z*_2_ with an unreliable timing. However, as it consists of attractor and transient dynamics (Fig. 8c, WM activity), the WM-network can process the information and sum both numbers up. The result *z*_3_ = *z*_1_ + *z*_2_ mod3 is the final result of the task. Note that, as in each phase the information relevant later on is stored in the LTM, the performance of the system is independent of the time span between the different phases and the WM can also perform other tasks in between. Furthermore, due to the different time scales and phases involved, both networks – WM and LTM – and their interaction are required to solve this multi-phase task.

The multi-phase task implies several sources of unreliability of input timings perturbing the proper function of the WM. External inputs can be unreliable (similar to Figs 1, 2, 3 and 4) as well as inputs from the LTM (Fig. 7). Even when the external signals are accurate in timing and the LTM is at each recall in the same initial state (here in the silent state; see Fig. 8c, LTM ass. activity), the context cue can induce unreliability. Namely, differences in the context cue triggering the recall of the corresponding association (third phase) compared to the original context signal presented during learning (first and second phase) yield a distribution of LTM recall timings with a significant standard deviation (*σ*_recall_ ≈ 28 ms, Fig. 8d). Already this cue-induced variation alone leads to a doubling of the error when using a purely transient network as WM (dashed line in Fig. 8e). All these different sources of unreliability together impede the proper function of purely transient networks to solve this task. Therefore, all our attempts to solve this task with such a purely transient network failed. This indicates that the dynamics underlying working memory should consist of a combination of transient and attractor dynamics.

## Discussion

The neuronal network dynamics underlying the proper function of working memory (WM) is still an unresolved question. Experimental findings are diverse with some studies supporting the view that WM operates mainly by transient dynamics^18, 19^ while others indicate that persistent activities, i.e. attractor states, suffices to explain WM functions^9, 10^. Here, we considered the *N*-back task with variances in the timing of input stimuli^22^ to draw conclusions on this dynamics. First, we showed that in purely transient systems the information about the *N* past stimuli is stored, as expected, in distinguishable trajectories. However, if the variance in the input timings increases, the trajectories are disturbed resulting in large overlaps between them which impede the readout of the stored information by downstream neurons. In contrast, introducing attractor states in the dynamics “structures” the phase space of the system: It stores the history of the past stimuli by remaining in the corresponding attractor. Only if a new stimulus is presented, independent of the timing, the system’s dynamics traverses by “tubes” of transient dynamics to another, history-dependent attractor. This phase of transient dynamics between the attractor states is sufficient to perform complex temporal computations.

The most common type of purely transient network models of WM are reservoir networks^5, 21, 25, 26, 46^. The robustness of their performance when confronted with noise in the input or within the network has been extensively studied^34, 47–50^. However, the susceptibility of such systems to variances in the timing of the input stimuli (Figs 1 and 2) has – to the best of our knowledge –not been considered and found before. Due to the universality of reservoir networks^25^, we expect that the here-presented findings can be generalized to a large class of purely transient systems implying that purely transient dynamics in general are inadequate to describe the dynamics underlying WM. Instead, a combination of transient and attractor dynamics is required (Figs 3 and 4).

Systems consisting of transient dynamics and attractor states have been proposed and investigated before^14, 37, 38^. In general, these attractors are formed by introducing additional readout neurons which feed back into the generator network. In fact, several studies^33–35^ indicate that a feedback from the readout neurons to the generator network enhances the stability and the performance of the network and can even lead to a recurrent neural network with universal computing power^34^. Additionally, the feedback can also be replaced by adapting the synaptic weights within the network in order to introduce attractor states^51^. Note that the attractor states do not have to be stable fixed points of the dynamics. It could be sufficient to introduce slow states^38^ or heteroclinic channels^4, 52^ to make the system robust against variances in input timings. Furthermore, given that neuronal systems have multiple possibilities to store information^53^, the attractors could also be realized in the synaptic dynamics^54, 55^ instead of (persistent) neuronal activities.

The here-derived hypothesis that the combination of transient dynamics and attractor states underlies the dynamics of WM is also supported by several experimental studies: For instance, it was shown that during a monkey WM-task, stimuli trigger the neuronal dynamics of prefrontal cortex neurons to perform fast (transient) transitions through different states until reaching a low-energy attractor state^56^. Similar dynamics are also found on larger brain scales in a fMRI study^57^. Interestingly, in the odor system of the locust, an odor stimulus evokes a succession of states resulting in an odor specific fixed point. However, the separation of different stimuli (a computational task) is optimal during the initial transient dynamics^4, 12^ supporting our hypothesis.

To solve more complex tasks, the WM has also to consider information stored in the long-term memory (LTM). The resulting interaction between WM and LTM is extensively investigated in psychological and psychophysical experiments^29–32^ and its neuronal implementation^2, 10, 58–60^. However, from the theoretical side, this interaction has not been studied intensively (but see refs 61 and 62). Here, we show that LTM-systems are unreliable in recall timings (Fig. 7) which implies that the WM-network has to be robust against these unreliabilities to functionally interact with the LTM (Fig. 8). This robustness is achieved by the combination of attractor and transient dynamics underlying working memory.

In summary, in the present study we consider the variance in timings of the inputs to draw conclusions about the underlying basic neuronal dynamics of WM. In particular, we show that a reliable WM-system requires a combination of attractor states and transient dynamics. Furthermore, we argue that these different dynamic regimes have also different functional roles – attractors store information while transients process information – enabling a continuous interaction with an adaptive LTM-system and yielding experimental verifiable predictions. This provides a further step in understanding the principles generating the functionally important dynamics of working memory.

## Methods

### Reservoir Network

The network consist of an input layer, a generator network, and readout neurons. Accordingly, neurons in the actual reservoir are named generator neurons. With *N*_G_ generator neurons, *N*_I_ input signals and *N*_R_ readout neurons, and, if any, *N*_A_ additional specially-trained neurons, the dynamics of the generator neuron *i* is given by a membrane potential ${u}_{i}^{{\rm{G}}}$ described by

1 $$\tau \frac{{\rm{d}}{u}_{i}^{{\rm{G}}}}{{\rm{d}}t}=-\,{u}_{i}^{{\rm{G}}}+\sum _{j=1}^{{N}_{{\rm{G}}}}{w}_{ij}^{{\rm{GG}}}{F}_{j}^{{\rm{G}}}+\sum _{k=1}^{{N}_{{\rm{I}}}}{w}_{ik}^{{\rm{GI}}}{I}_{k}+\sum _{l=1}^{{N}_{{\rm{R}}}}{w}_{il}^{{\rm{GR}}}{R}_{l}(+\sum _{m=1}^{{N}_{{\rm{A}}}}{w}_{im}^{{\rm{GA}}}{A}_{m})$$

with time constant *τ*, firing rates ${F}_{j}^{{\rm{G}}}=\,\tanh ({u}_{j}^{{\rm{G}}})$ of generator neurons, input signals *I*_*k*_, readout signals *R*_*l*_ and, if present, specially-trained additional readouts *A*_*m*_. The synaptic weights ${w}_{ij}^{{\rm{GG}}}$ within the generator network are drawn from a normal distribution with zero mean and variance ${g}_{{\rm{GG}}}^{2}/{N}_{{\rm{G}}}$. Similarly, the synaptic weight ${w}_{il}^{{\rm{GR}}}$ from the readout neuron *l* back to the generator neuron *i* is drawn from a normal distribution with zero mean and variance ${g}_{{\rm{GR}}}^{2}/{N}_{{\rm{R}}}$ and the weight ${w}_{im}^{{\rm{AR}}}$ from specially-trained neuron *m* is drawn from a normal distribution with zero mean and variance ${g}_{{\rm{GA}}}^{2}/{N}_{{\rm{A}}}$. Every generator neuron *i* receives signals from exactly one randomly chosen input signal *k* scaled by a weight ${w}_{ik}^{{\rm{GI}}}$ drawn from a normal distribution with variance ${g}_{{\rm{GI}}}^{2}$ and zero mean.

The current activity value *R*_*l*_ of the linear readout neuron *l* = 1, …, *N*_R_ is given by

2 $${R}_{l}=\sum _{i=1}^{{N}_{{\rm{G}}}}{w}_{li}^{{\rm{RG}}}{F}_{i}^{{\rm{G}}}\mathrm{.}$$

The initial values of the weights ${w}_{li}^{{\rm{RG}}}$ are drawn from a normal distribution with zero mean and variance ${N}_{{\rm{G}}}^{-1}$. These weights are adapted by different supervised algorithms described below. If there are any specially-trained additional readout units in the network, they follow identical dynamics as the default readout neurons.

If not stated otherwise, the used parameter values are *τ* = 10 ms, *N*_*G*_ = 100, *N*_*I*_ = 1, *N*_*R*_ = 1, *g*_GG_ = 1.0, *g*_GI_ = 1.0. All equations are solved by using the Euler method with a time step of $\hat{t}=1\,{\rm{ms}}$.

#### Echo State Network Approach

Following the echo state network (ESN) approach^25^ to train the weights from the reservoir network to the readouts ${w}_{li}^{{\rm{RG}}}$, the network is sampled for a given number *S* of time steps. The activities of the generator neurons are collected in a state matrix *M* of dimension *N*_G_ × *S* with every row containing the activities at a specific time step. The corresponding target signals of the readout neurons are collected in a teacher matrix *T* of dimension *S* × *N*_R_. Optimizing the mean squared error of the readout signals is achieved by calculating the pseudoinverse *M* ^−1^ of *M* and setting the weight matrix accordingly:

3 $${W}^{{\rm{RG}}}={M}^{-1}\,T\mathrm{.}$$

Note that during the sampling phase, instead of the actual activities of the readout neurons the values of the target signals modified by Gaussian noise with variance ${\sigma }_{{\rm{noise}}}^{2}$ are fed back to the generator network. We use *σ*_noise_ = 0.1.

#### FORCE Approach

In contrast to the ESN approach, FORCE learning^33^ is an online-learning method. As originally proposed, we utilize the recursive least-squares (RLS) algorithm^63^ to adapt the readout weights fast enough to keep the actual activities of the readout neurons close to the target values from the very beginning. During learning, in every simulation step, the readout weight vector for readout neuron *l* at time *t* is adapted according to

4 $${w}_{l}^{{\rm{RG}}}(t)={w}_{l}^{{\rm{RG}}}(t-\hat{t})-{e}_{l}(t)(P(t){F}^{{\rm{G}}}(t{))}^{T}\mathrm{.}$$

Here, $\hat{t}$ denotes the step width of the simulation and

5 $${e}_{l}(t)={({w}_{l}^{{\rm{RG}}}(t-\hat{t}))}^{T}{F}^{{\rm{G}}}(t)-{f}_{l}(t)$$

is the difference between the readout signal and the target function *f*_*l*_(*t*). The matrix *P*(*t*) has the dimension *N*_G_ × *N*_G_ and is updated according to

6 $$P(t)=P(t-{\rm{\Delta }}t)-\frac{P(t-\hat{t}){F}^{{\rm{G}}}(t){F}^{{\rm{G}}}{(t)}^{T}P(t-\hat{t})}{1+{F}^{{\rm{G}}}{(t)}^{T}P(t-\hat{t}){F}^{{\rm{G}}}(t)}$$

with an initial value of *P*(0) = *α* ^−1^𝟙 where 𝟙 is the identity matrix. We set *α* = 100.

### Benchmark Task

For the benchmark task (Fig. 1), input pulses are generated at random time intervals drawn from a normal distribution with mean *μ*_Δ*t*_ and variance *σ*_Δ*t*_. Every pulse is modeled as a convolution of a constant signal with length *t*_pulse_, unit magnitude and random sign and a Gaussian window with variance ${\sigma }_{{\rm{smooth}}}^{2}$. To avoid overlaps between pulses, we restrict the time interval between two pulses to a minimum of 2 ⋅ *t*_pulse_. The target readout signal consists of pulses of identical shape whose signs depend on the sign of the respective second-last input pulse. To account for the delays within the generator network, each target pulse is generated with a short delay *t*_delay_ after the corresponding input pulse.

In the case with specially-trained neurons (or additional readout units) (Fig. 3), we add two further target signals. These signals have a value of either +1 or −1 with the sign depending on the sign of the last or second-last input pulse, respectively. Also here, the pulses start with a short delay *t*_delay_ with respect to the input pulses and they are smoothed by a convolution with a Gaussian window with variance ${\sigma }_{{\rm{smooth}}}^{2}$.

To evaluate the performance of the network in generating the desired target signal *f*_*l*_(*t*) at the read out neuron *l*, we compare the actual activity *R*_*l*_(*t*) with the desired target signal *f*_*l*_(*t*) and calculate the root mean square error (RMS). In order to normalize this value, it is divided by the RMS of the target signal:

7 $${E}_{l}=\frac{\sqrt{{\sum }_{t}{({R}_{l}(t)-{f}_{l}(t))}^{2}}}{\sqrt{{\sum }_{t}\,{f}_{l}{(t)}^{2}}},\,l=1,\ldots ,{N}_{{\rm{R}}}\mathrm{.}$$

For the benchmark task, there is only one regular readout unit (*l* = 0). We therefore omit the index such that *E* = *E*_0_. Every parameter configuration is evaluated in 1000 independent network instantiations.

When adding the recall stimulus in the benchmark task (Fig. 6), it is applied 10 ms before the onset of the target pulse via an additional input neuron (*N*_*I*_ = 2) and given by a short positive unit pulse of length *t*_pulse_ smoothed by a convolution with a Gaussian window with variance ${\sigma }_{{\rm{smooth}}}^{2}$.

The used values are *μ*_Δ*t*_ = 200 ms, *t*_pulse_ = 10 ms, *t*_delay_ = 10 ms, and *σ*_smooth_ = 2 ms.

### Hopfield Network

We use a standard implementation of the Hopfield network^43^. It consists of *N*_Hopf_ = 1000 binary neurons. In every time step, every neuron *i* is in one of two possible states characterized by a firing rate of either *F*_*i*_ = 1 or *F*_*i*_ = −1. The activity states of all neurons are updated according to

8 $${F}_{i}(t+\mathrm{1)}=(\begin{array}{cc}\mathrm{1,} & {\rm{if}}\sum _{j=1}^{{N}_{{\rm{Hopf}}}}{w}_{ij}^{{\rm{Hopf}}}{F}_{j}(t)\ge 0\\ -\,\mathrm{1,} & {\rm{if}}\sum _{j=1}^{{N}_{{\rm{Hopf}}}}{w}_{ij}^{{\rm{Hopf}}}{F}_{j}(t) < \mathrm{0,}\end{array}$$

where ${w}_{ij}^{{\rm{Hopf}}}$ is the synaptic weight projecting from neuron *j* to neuron *i* (w.l.o.g., ${w}_{ii}^{{\rm{Hopf}}}=0$). We store *K* = 100 random patterns ${\hat{F}}_{j}$ into the network by choosing

9 $${w}_{ij}^{{\rm{Hopf}}}=\frac{1}{K}\sum _{\mu }^{K}{\hat{F}}_{i}^{\mu }{\hat{F}}_{j}^{\mu }\mathrm{.}$$

The network is initialized with a binary vector *F* ^0^ whose entries differ in *d* positions from ${\hat{F}}^{1}$ corresponding to an overlap of 1 − *d*/*N*_Hopf_. We simulate the development of the network until a stable pattern is reached. Hence, the number of simulated time steps corresponds to the convergence time. In a few cases, the system does not a reach a stable pattern. The respective trials are excluded from the analysis. For every distance *d* with 0 ≤ *d* ≤ *N*, we repeated the simulation 10,000 times.

### Self-Organized Cell Assemblies

The here-used LTM-model is based on Hebbian cell assembly models^28^. We introduce some modifications to allow for a self-organized allocation of memory representations. The main part of the model is an *n* × *n*-grid of excitatory neurons, each described by a membrane potential *u*_*i*_, *i* = 1, …, *N*_CA_ (*N*_CA_ = *n* ^2^), according to

10 $$\tau \frac{{\rm{d}}{u}_{i}}{{\rm{d}}t}=-\,{u}_{i}+\sum _{j\mathrm{=1}}^{{N}_{{\rm{CA}}}}{w}_{ij}^{{\rm{lat}}}{F}_{j}+{w}_{{\rm{exc}},{\rm{inh}}}{F}^{{\rm{inh}}}+\sum _{k\mathrm{=1}}^{{N}_{{\rm{aff}}}}{w}_{ik}^{{\rm{aff}}}{F}_{k}^{{\rm{aff}}}$$

with time constant *τ* (the same value as for the time constant of the reservoir neurons). *F*_*i*_ represents the firing rate of neuron *i* given by

11 $${F}_{i}=\frac{1}{1+\exp (\beta (\varepsilon -{u}_{i}))}$$

with parameters *β* and *ε*. Every neuron *i* on the grid receives lateral inputs with synaptic weights ${w}_{ij}^{{\rm{lat}}}$ from all grid neurons *j* whose euclidean distance from neuron *i* (measured in grid units) is smaller than the excitatory interaction radius *r*_exc_ (otherwise ${w}_{ij}^{{\rm{lat}}}=0$). In order to avoid boundary effects, periodic boundary conditions are applied.

In addition to the excitatory neurons, we consider an inhibitory population which receives signals from all excitatory grid neurons and projects signals back to all of them. The mean-field dynamics of the membrane potential *u* ^inh^ and the firing rate *F* ^inh^ of this population is given by

12 $${\tau }_{{\rm{inh}}}\frac{{\rm{d}}{u}^{{\rm{inh}}}}{{\rm{d}}t}=-\,{u}^{{\rm{inh}}}+\sum _{i=1}^{{N}_{{\rm{CA}}}}{w}_{{\rm{inh}},{\rm{exc}}}{F}_{i}$$

and

13 $${F}^{{\rm{inh}}}=\frac{1}{1+\exp ({\beta }_{{\rm{inh}}}({\varepsilon }_{{\rm{inh}}}-{u}^{{\rm{inh}}}))}\mathrm{.}$$

Here, *w*_inh,exc_ and *w*_exc,inh_ designate the average synaptic weight from excitatory neurons to the inhibitory population and vice versa.

A second layer of *N*_aff_ afferent neurons projects signals onto the grid layer via afferent synapses with weights ${w}_{ik}^{{\rm{aff}}}$. The firing rates ${F}_{k}^{{\rm{aff}}}$ of these neurons are externally controlled and represent the input pattern presented to the network. Every grid neuron *i* receives synapses from exactly *n*_aff_ randomly chosen input neurons.

Nonzero lateral synapses as well as the nonzero afferent synapses are governed by a synaptic plasticity rule composed of a Hebbian term, correlating the postsynaptic activity *F*_*i*_ and the presynaptic activity *F*_*j*_, and a synaptic scaling term, driving the postsynaptic activity towards a target value *F*_*T*_^28, 64^:

14 $${\tau }_{w}\frac{{\rm{d}}{w}_{ij}}{{\rm{d}}t}={F}_{i}{F}_{j}+({F}_{T}-{F}_{i}){w}_{ij}^{2}\mathrm{.}$$

To obtain the memory convergence times for this model (Fig. 7d), we consider several phases: In the first phase, the learning phase, we fully activate half of the afferent neurons (representing an input pattern) and simulate the network long enough to obtain a stable cell assembly (memory representation) in the grid layer. All neurons with a firing rate *F*_*i*_ larger than 0.5 are considered to be part of the assembly. In the next phase, we deactivate the input to assure that the resulting network structures do not imply persistent activities. In the recall phase, a similar but slightly different input pattern where *d*/2 previously active afferent neurons are now inactive and *d*/2 originally inactive afferent neurons are now active is presented to the network. This corresponds to a total pattern difference of *d* and therefore to an overlap of 1 − *d*/*N*_aff_. Applying the modified afferent pattern, we measure the time the network requires until 90% of the neurons which have been already active during learning (representing the stored pattern) get active. The respective time interval is considered as the convergence time of the stored memory. For every difference *d* with 0 < *d* < *N*/2 this procedure is repeated for 1000 times. Trials during which 90% activation is not reached are not considered for further analyses.

The used parameters values are *n* = 30 (*N*_CA_ = 900), *τ* = 0.01 s, *w*_exc,inh_ = −20.0, *β* = 1, *ε* = 12, *r*_exc_ = 3, *τ*_inh_ = 0.02 s, *w*_inh,exc_ = 1.0, *β*_inh_ = 0.1, *ε*_inh_ = 100, *N*_aff_ = 50, *n*_aff_ = 20, *τ*_*w*_ = 10 s, and *F*_*T*_ = 0.

### WM-LTM interaction

For the interaction of WM with LTM, as sketched in Fig. 8, we trained a reservoir network (*N*_*G*_ = 500) with *N*_*I*_ = 4 input signals (“0”, “1”, “2”, “+”) and *N*_*R*_ = 9 readouts. The first three of these readouts represent the actual output of the WM (“0”, “1”, “2”). The second group of three holds additional assisting outputs which encode the last received number input. Accordingly, the third group encodes the second-last received number input. Already during the reservoir training, the actual readouts of the reservoir are connected to the LTM-symbol area. For simplicity, this area is modeled as a collection of single excitatory neurons with dynamics and parameters as described above. Every number is represented by a group of ten neurons, the two context signals by twenty neurons each. The reservoir output signals are fed into the respective symbol neurons with strong synaptic weights *w* ^SR^ = 20. The activities of the symbol neurons, in turn, are directly taken as input signals for the reservoir.

The reservoir network is trained using the FORCE approach as described above. All inputs are smoothed by convolution with a Gaussian kernel with a standard deviation of *σ*_smooth_ = 5 ms. The pulses representing numbers have a length drawn from a uniform distribution with a minimum of *t*_num,min_ = 50 ms and a maximum of *t*_num,max_ = 100 ms. The pulses representing the “+”-operation have a length uniformly drawn between *t*_+,min_ = 100 ms and *t*_+,max_ = 400 ms. The distances between the individual pulses vary between Δ*t*_min_ = 100 ms and Δ*t*_max_ = 400 ms. The reservoir network is trained for 100000 time steps and, afterwards, its performance is evaluated for the same number of time steps. This procedure is repeated until the normalized error during evaluation drops below 0.05.

After training the reservoir network, the LTM-symbol area is taken as an input to the LTM-association area which is modeled by the system of self-organized cell assemblies described above. Apart from the higher number of afferent synapses (*n*_aff_ = 30) per excitatory grid neuron, we use the same parameters as before. However, in order to be able to recall actual symbols from the cell assembly activity, additional feedback synapses from the LTM-association area back to the symbol area are introduced. These are governed by the same synaptic plasticity processes as the lateral and afferent synapses. Every neuron in the symbol area receives synapses from *n*_fb_ = 250 randomly chosen LTM-association neurons. The feedback synapses are initialized with a weight which equals zero.

## Electronic supplementary material

Supplementary Material
