# Supplementary material for: Working Memory Requires a Combination of Transient and Attractor-Dominated Dynamics to Process Unreliably Timed Inputs
Source: Sci Rep. 2017 May 30;7:2473. doi: 10.1038/s41598-017-02471-z (PMC5449410; doi:10.1038/s41598-017-02471-z)
Supplement: Supplementary file 1 — Supplementary Material [file 41598_2017_2471_MOESM1_ESM.pdf]

Supplementary Material: Working Memory Requires a Combination of Transient and Attractor-Dominated Dynamics to Process Unreliably Timed Inputs

Timo Nachstedt[1,2,*] **and Christian Tetzlaff**[2,3]

[1]Third Institute of Physics, Universität Göttingen, 37077 Göttingen, Germany
[2]Bernstein Center for Computational Neuroscience, 37077 Göttingen, Germany
[3]Max Planck Institute for Dynamics and Self-Organization, 37077 Göttingen, Germany
[*]timo.nachstedt@phys.uni-goettingen.de

Supplementary Figures

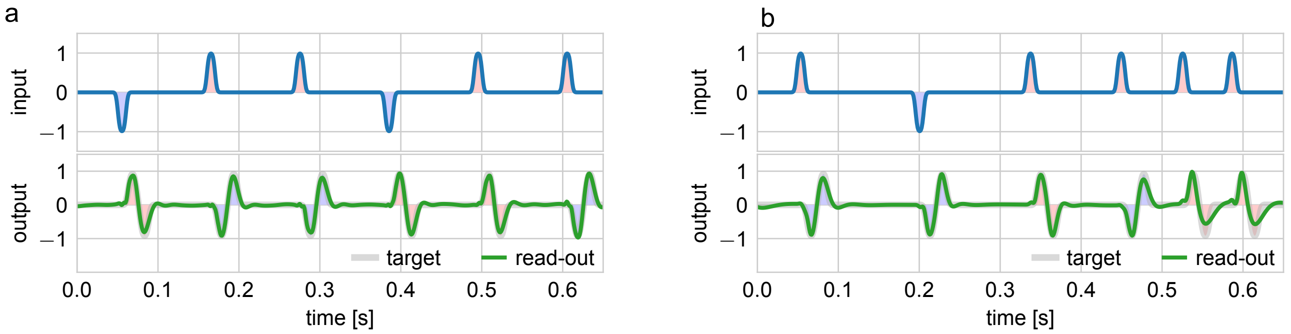

Supplementary Figure S1. Sine-shape readout signal. Instead of the pulse-shaped readout signal, the reservoir can be trained to produce also more complex target signals when performing the benchmark task used throughout the publication. For both plots, a network with $N_G = 100$ generator neurons was trained using the ESN approach. **(a)** Reservoir network without additional readouts and with no feedback ($g_{gr} = 0$) operating on an input signal without input timing variance ($\sigma_{\Delta t} = 0\,\text{ms}$). **(b)** Reservoir network with additional readouts providing strong feedback ($g_{ga} = 1.0$) trained and operating on an input signal with input timing variance $\sigma_{\Delta t} = 50\,\text{ms}$.

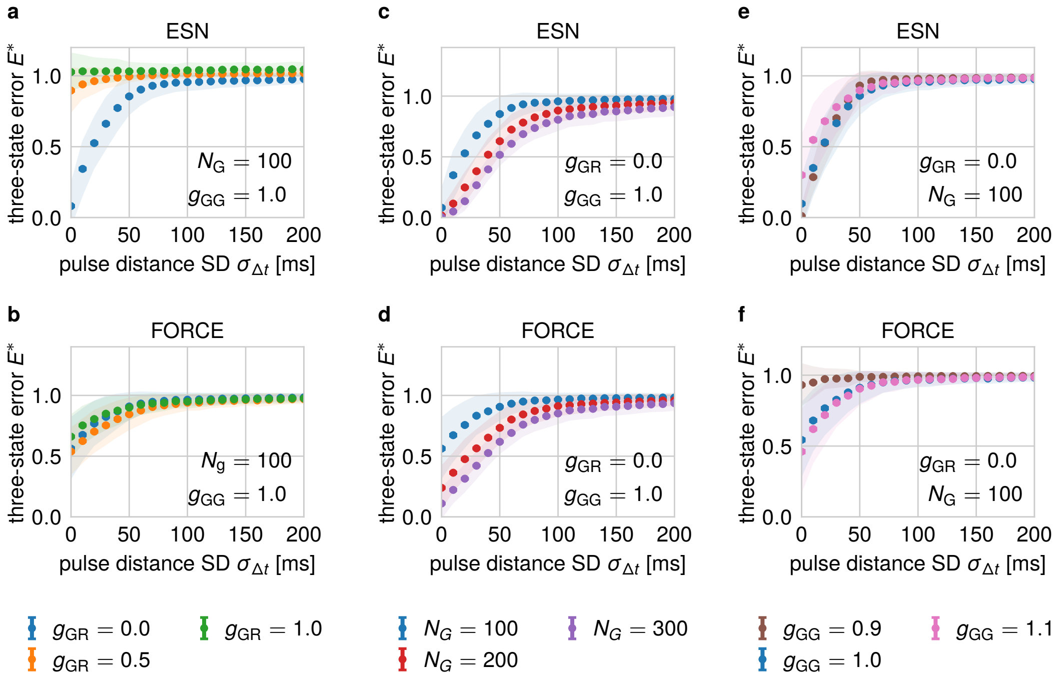

Supplementary Figure S2. N-back performance of the transient network for a three-state error evaluation. For calculating the three-state error E^*, we consider discretized signals $R^*(t)$ and $f^*(t)$ of the readout signal $R(t)$ and the target signals $f(t)$. In particular, we set $R^*(t) = -1$ if $R(t) < -0.5$, $R^*(t) = 0$ if $-0.5 < R(t) < 0.5$, and $R^*(t) = 1$ if $0.5 < R(t)$. Accordingly, we have $f^*(t) = -1$ if $f(t) < -0.5$, $f^*(t) = 0$ if $-0.5 < f(t) < 0.5$, and $f^*(t) = 1$ if $0.5 < f(t)$. Similar to the original error, we then define the error E^* as the ratio of the RMS of $R^*(t) - f^*(t)$ and the RMS of $f^*(t)$. The underlying data is the same as in Figure 2. **(a), (b)** The network is trained with three different values of the standard deviation g_{GR} of the feedback-weights from the readout neurons to the generator network. The constant parameters are $N_G = 100$ and $g_{gg} = 1.0$. **(c), (d)** Networks of different sizes, i.e. different values of N_G, are trained to perform the benchmark task. The constant parameters are $g_{GR} = 0$ and $g_{gg} = 1.0$. **(e), (f)** The influence of different values g_{GG} of the internal weights of the generator network is investigated. The constant parameters are $g_{GR} = 0$ and $N_G = 100$.

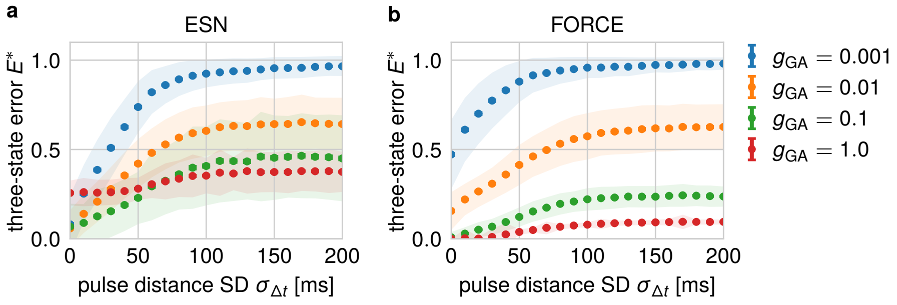

Supplementary Figure S3. N-back performance of the network with specially-trained readout signals for a three-state error evaluation. The three-state error E^* is determined as outlined in Supplementary Figure S2. The underlying data is the same as in Figure 4. **(a)** ESN approach; **(b)** FORCE-method.

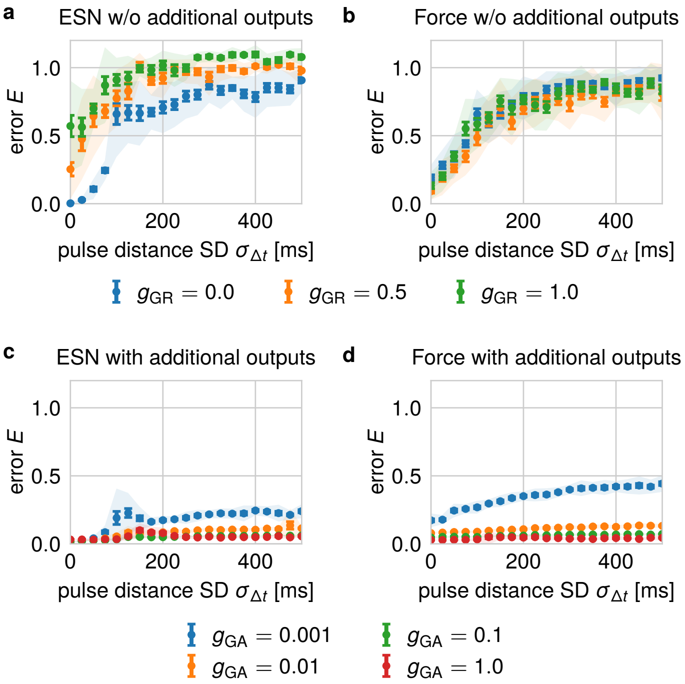

Supplementary Figure S4. N-back task results for a reservoir with $N = 1000$ neurons. As found for smaller reservoirs in Fig. 2, the introduction of variance in the input timing rapidly increases the the mean normalized readout error E also for significantly larger purely transient reservoir networks. Also similar to smaller networks (Fig. 4), introducing feedback from specially-trained neurons renders the network resistant to variance in the input timing. Every data point represents the mean of 20 network instantiations. If in one instantiation the error after training is larger than 1.5, we consider the respective training procedure as not converged and exclude it from the mean. The mean of the interstimulus intervals of the input signal is $\mu_{\Delta t} = 0.5\,\mathrm{s}$. **(a)** Network trained using the echo state network approach (ESN) without additional specially-trained neurons. Three different values of the standard deviation g_{GR} of the feedback-weights from the readout neurons to the generator network are employed. **(b)** As (a), but the network is trained using the FORCE-learning method. **(c)** Network trained using the ESN approach with different strength of the feedback from additional specially-trained neurons. **(d)** As (c), but the network is trained using the FORCE-learning method.

Supplementary Discussion S1: Dynamics in the reservoir with specially-trained neurons

First, we discuss the example trajectory in Fig. 5a. We assume that the network has just received a positive stimulus, the last one has been negative and the second last one positive. In this case, the network dynamics flows along the red trajectory marked by $*_1$. If the next stimulus has a negative sign, the dynamics takes at the fork (magnification in left inset) the right, blue trajectory ($*_2$). Along this trajectory the downstream neurons are able to read out the stored negative sign (second last stimulus) and to produce the corresponding target shape at the output. When the network dynamics reaches the next fork (magnification in right inset), we assume the network receives again a negative pulse. Thus, the dynamics follow the right, red trajectory ($*_3$) and the positive sign can be read out (now second last input). The dynamics reaches the other fork magnified in the right inset and so on. Thus, the information about the signs of the received stimuli is stored in the trajectory the network takes (transient dynamics).

Extending the reservoir network by the specifically trained neurons changes the dynamics of the system significantly (here, $g_{\mathrm{GA}} = 1$): The network now possesses four distinct attractor states with specific, transient trajectories interlinking them (Fig. 5c). The same example of stimuli as above is now processed in the system as follows: The last two stimuli to the network have been negative and positive such that the network dynamics is in the attractor state marked by $\diamond_1$. Now, the new negative stimulus drives the dynamics along the blue trajectory ($\diamond_{21}$) to the next attractor state ($\diamond_2$). As long as the dynamics is on the trajectory, downstream neurons can read out the stored information (negative sign) and the corresponding output target shape. Given the next stimulus (negative), the network dynamics follow another trajectory and the stored positive sign can be read out ($\diamond_{32}$) before the network dynamics reaches the next attractor state ($\diamond_3$). Thus, the information about the sign of the two last stimuli is stored in the attractor states while the transients, connecting them, are used to process the information and to produce the complex output signal (target shape).
